# Outbreak of *Trypanosoma vivax* in Dairy Cows: Hematologic, Immunological and Antioxidant Responses Before and After Treatment with Isometamidium Chloride

**DOI:** 10.3390/pathogens14020143

**Published:** 2025-02-04

**Authors:** Alexandro Fritzen, Maksuel Gatto de Vitt, Guilherme Luiz Deolindo, Mateus Henrique Signor, Nathalia Gemelli Correa, Brenda Guedes Ribeiro, Julia Marques, Gabriella Bassi das Neves, Luiz Claudio Miletti, Aleksandro Schafer da Silva

**Affiliations:** 1 Graduate Program in Biochemistry and Molecular Biology, Universidade do Estado de Santa Catarina (UDESC), Lages 88520-000, Brazil; vet.fritzen@gmail.com (A.F.); guilhermeluizd@outlook.com (G.L.D.); brenda.gr1201@edu.udesc.br (B.G.R.); julia.marques17@edu.udesc.br (J.M.); gabriella.neves06@edu.udesc.br (G.B.d.N.); luiz.miletti@udesc.br (L.C.M.); 2 Graduate Program of Animal Science, Universidade do Estado de Santa Catarina (UDESC), Chapecó 89815-630, Brazil; mak-witt@hotmail.com (M.G.d.V.); mateushenriquesignor@gmail.com (M.H.S.); 3 Department of Animal Science, Universidade do Estado de Santa Catarina (UDESC), Chapecó 89815-630, Brazil; natalia.gc@outlook.com

**Keywords:** *Trypanosoma vivax*, immunosuppression, cattle, dairy

## Abstract

*Trypanosoma vivax* infection is an emerging condition that causes damage and mortality among cattle and is transmitted by mechanical vectors or contaminated fomites. This disease has been spreading in southern Brazil, causing anemia, weight loss, diarrhea, abortion, and infertility; however, its behavior and host–parasite relationships are not yet fully understood. To clarify this issue, animals that presented clinical signs were subjected to an immunochromatographic screening test. An indirect immunofluorescence test was then performed on samples collected before treatment (the gold standard test), which showed that in the herd of 20 cows, we had 14 seropositive for *T. vivax*. Blood samples were collected before and after treatment to study the effects of the disease and treatment, with the cows divided into two groups: infected and uninfected. Cows were evaluated for hematologic, biochemical, and antioxidant responses, comparing them with uninfected and infected animals, as well as pre- and post-treatment (isometamidium chloride—1 mg/kg body weight [BW]). There was no difference (*p* > 0.05) between groups in milk production and feed intake; however, ten days after treatment, there was an increase of 1.72 kg of milk in cows diagnosed as infected with *T. vivax*. Seropositive cows had lower erythrocyte counts, hemoglobin concentrations, hematocrit, platelet counts, and lymphocyte and granulocyte counts. In seropositive cows, the higher total protein concentration is due to increased globulins, which the protein profile by electrophoresis showed to be related to higher levels of immunoglobulins (IgA and other heavy-chain immunoglobulins), ceruloplasmin, haptoglobin, ferritin, C-reactive protein; associated with lower transferrin levels. The activity of the enzymes aspartate aminotransferase, gamma-glutamyl transferase, cholinesterases, and creatine kinase were compared in seronegative and seropositive cows for *T. vivax*. Lower serum calcium levels were observed in seropositive cows. Cows diagnosed with trypanosomosis presented high levels of reactive oxygen species, lipid peroxidation, protein oxidation, nitrite/nitrate activity, superoxide dismutase, and glutathione peroxidase. The enzymes catalase and glutathione S-transferase presented lower activity in the blood of seropositive cows compared to the control on the day of diagnosis, which was no longer observed ten days after treatment. The animals exhibited hypogalactia, anemia, thrombocytopenia, leukopenia, and acute phase response accompanied by liver and muscle tissue damage and oxidative stress, demonstrating the effect of *T. vivax* infection in naturally infected Jersey cows.

## 1. Introduction

Bovine trypanosomosis, caused by *Trypanosoma vivax*, is spreading in Brazil [1]. First diagnosed in this country in 1972, it has since emerged in several states [2], including regions in the south, such as Santa Catarina and Rio Grande do Sul [3]. Trypanosomiasis is an arthropod-borne infection, vectored by flies of the genus *Glossina* in Africa. At the same time, its transmission in South America is attributed to contaminated fomites or mechanical vectors such as tabanids. However, experimental transmission by *Stomoxys calcitrans* flies has not been successful [4], generating new possibilities concerning transmission routes in the American condition. After inoculation into the skin, the parasite grows for a few days, enters the lymph nodes and later the blood, where it reproduces by binary fission and can lead to peaks of parasitemia that are accompanied by clinical signs such as depression, fever, anorexia, weight loss, anemia, edema, and nervous signs, with spontaneous abortion and diarrhea observed in some cases [5].

Animals infected with *T vivax* present with varied clinical conditions, ranging from severe hemorrhages accompanied by death to subclinical presentations with the presence of antibodies and oscillating parasitemia accompanied by immunosuppression and concomitant diseases caused by other agents [6]. The effect of the parasite on the vascular bed is marked, leading to the formation of thrombi, vascular damage, and neurological lesions as meningoencephalitis, resulting from vascular and autoimmune effects and the formation of immune complexes consequences from the interaction of the immune system with the parasite [7]. Trypanosomosis can be diagnosed using an indirect immunofluorescence test, which looks for animals with seroconversion. This test is the gold standard for this disease due to intermittent parasitemia, leading to false negatives in tests that look for the parasite in the blood (PCR, for example) [3]. In turn, treatment is carried out in Brazil using dimenazene diaceturate at 7.0 mg/kg and isometamidium at 0.5 and 1.0 mg/kg, showing high efficiency in controlling and eliminating parasitemia [8].

Studies with experimental infection of cattle demonstrate the extravascular presence of *T. vivax* DNA in tissues in the acute and chronic phases of infection, increasing the evidence that *T. vivax* is not exclusively a blood parasite [9]. The detection of the parasite in tissues for long periods makes the idea of “self-cure” little applicable in this scenario and makes treatment difficult [9,10]. These differences lead to the need to understand the characteristics of bovine trypanosomosis and the effect of treatment on the hematologic, metabolic, and oxidative parameters of naturally infected cows before and after treatment with isometamidium.

## 2. Materials and Methods

### 2.1. History

In January 2023, 70% of cows and all pregnant heifers (seven animals) were sick, with the laboratory-clinical-therapeutic diagnosis of babesiosis and anaplasmosis (etiological agents visualized on a slide). Among the sick animals, three died from severe anemia (erythrocytes (mean 2.56 × 10^6^/µL), hemoglobin (mean 6.20 mg/dL), and hematocrit (between 12.5%). The remaining animals were medicated (diminazene aceturate 3.5 mg/kg BW, oxytetracycline 10 mg/kg BW, antitoxic (Mercepton^®^, Laboratório Bravet, Rio de Janeiro Brazil—50 mL/animal), injectable iron (4 mL/animal for four consecutive days) and vitamin supplement B12 (5 mL/animal in three doses at 48 h intervals); they recovered. However, eight of these animals had relapses of hyperthermia (controlled with dipyrone) and mild anemia (verified by complete blood count), reducing feed intake and milk production between April and May 2023. Once these clinical signs were observed, therapy was carried out with a single dose of imidocarb dipropionate (1.8 mg/kg BW); the animals improved, and the anemia remained mild: erythrocytes (between 4 to 5 × 10^6^/µL), hemoglobin (between 7.8 to 9.5 mg/dL), and hematocrit (between 18.6 to 23.7%). Blood smears from the cows were taken and stained with the Panótico Rapido kit (Digilab, Piracicaba, Brazil) and tested negative for structures compatible with the *Anaplasma marginale* and *Babesia* spp. in microscopic analysis. In June 2023, another cow became sick and was medicated with an imidocarb dipropionate protocol, but it did not improve. It was necessary to perform a blood transfusion twice due to anemia (hematocrit of <12%). In the blood smear of this animal, a morphological structure compatible with trypomastigote was observed. Due to the high and recent incidence of *Trypanosoma vivax* in the west of Santa Catarina [3], it was suspected that this parasite infected cows. Therefore, we used a rapid, commercial diagnostic kit (Imunotest^®^, Imunodot Diagnostico Avançado, Jaboticabal, Brazil) for five cows in the herd, of which three were positive for *T. vivax*.

Then, a specific medication was applied to control *T. vivax* based on isometamidium chloride (Vivendium^®^, Ceva Saúde Animal Ltda, Paulínia, Brazil) in a single intramuscular injection of 1.0 mg/kg of body weight in all cows. We combined the treatment with an antitoxic dose of 40 mL/animal (intramuscular) (Mercepton^®^, Laboratório Bravet, Rio de Janeiro, Brazil). After 1 h of application, adverse reactions were observed in the cows, which became restless (lying down/getting up), rolling around on the sawdust bed in the shed, and reducing feed consumption. After six hours of application, no evident clinical changes in the cows were observed.

### 2.2. Animals, Diet, and Installation

The herd of 20 primiparous Jersey cows was included in this study. The animals are part of the experimental farm at the State University of Santa Catarina (UDESC) in Guatambu, southern Brazil. The animals are kept in a Compost barn, with a robotic milking system with guided flow, at 5:00 a.m. and 3:00 p.m. The cows were fed in individual feeders (corn silage and concentrate), in the robot during milking (robot feed), and in oat and ryegrass pasture for 3 h per day. It is estimated that food consumption was 15 kg DM/cow/day, 10.5 kg in the shed in a controlled manner, and 4.5 kg of pasture.

### 2.3. Serology Diagnostic and Experimental Design

#### 2.3.1. Indirect Immunofluorescence Assay-IFA

The immunofluorescence testing to detect anti-*T. vivax* antibodies was performed according to the methodology described by Cuglovici et al. [11] using as a cut-off the dilution 1:80. Briefly, the slides with fixed antigens were removed from the freezer and dried at room temperature. The positive (immune sheep serum for *T. vivax*) and negative (pre-immune serum) were diluted in phosphate-buffered saline (PBS) in 1:80 dilution. Field samples were diluted in PBS at 1:40, 1:80, and 1:160. Twenty μL of diluted serum were distributed on the slides containing the *T. vivax* antigen. The slides were incubated for 30 min in a humid chamber at 37 °C and washed with PBS thrice. The FITC-labeled anti-bovine conjugate was added to each slide at 1:300 dilution in PBS. The slides were again incubated for 30 min and washed with PBS as previously described. Dried slides were analyzed using a Nikon^®^ epifluorescent microscope (Microscope Central, Feasterville, PA, USA). After analysis, it was found that fourteen cows (14/20) were seropositive for *T. vivax*.

#### 2.3.2. Experimental Design

Two groups (Positive [n = 14] and Negative [n = 6]) were formed to evaluate the impacts of the disease on cows, as well as post-treatment effects.

### 2.4. Samples Collection

Blood samples were collected for hematologic, biochemical, and immunological analyses on days 1 (data from diagnosis and before treatment) and day 10 post-treatment. Collections were made through the coccygeal vein with needles and vacuolated tubes. Tubes with EDTA were used to prevent clotting and enable hematologic analysis, and another contained a clot activator to obtain serum. For serum separation, the tubes without anticoagulant were centrifuged at 3500 RPM for ten min, and the serum was transferred to identified microtubes and stored at −20 °C until further analyses were performed.

### 2.5. Milk Production and Feed Intake

Milk production data were obtained from the robotic milking system, as the amount of milk was measured every time the animals visited the robot. The amount of feed consumed by bovines in the warehouse was measured by the robot and in individual feeders, except for pasture intake.

### 2.6. Hemogram

Hematologic analyses were performed on Equipe Vet 3000^®^ automatic equipment (Equip, Itatiba, Brazil) using fresh blood. The equipment made it possible to quantify the total number of leukocytes and the differential (lymphocytes, granulocytes, and monocytes) total erythrocyte count, hemoglobin concentration, and hematocrit.

### 2.7. Serum Biochemistry

All variables were quantified using an EXC200 biochemistry analyzer (Zybio, Belo Horizonte, Brazil), automatic equipment. In the biochemical analyses, total protein, glucose, albumin, cholesterol, and urea serum levels were measured using commercial kits Analisa^®^ (Gold Analisa Diagnóstica Ltda, Belo Horizonte, Brazil). The levels of globulins were obtained using a mathematical calculation (total proteins—albumin). The activity of gamma-glutamyltransferase (GGT), aspartate aminotransferase (AST), cholinesterase, and creatine kinase were measured using Analisa commercial kits, following the kit manufacturer’s instructions.

### 2.8. Proteinogram

For protein fractionation, sodium dodecyl sulfate-polyacrylamide gel electrophoresis was performed, according to Tomasi et al. [12] using a mini gel (10 × 10 cm). The gel was stained with Coomassie Blue and photographed to identify and quantify the protein fractions using Labimage1D software, L340 number (Loccus Biotechnology, Cotia, Brazil). A standard containing fractions with molecular weight between 10 and 250 KD (Kaleidoscope—Bio-Rad Laboratories, São Paulo, Brazil) was used as a reference.

### 2.9. Oxidative Stress

First, protein content was measured following the protocol established by Read and Northcote [13] using bovine serum albumin as the standard. Then, protein levels were adjusted, and the analysis described below began.

Lipid peroxidation was determined as TBARS levels using the methods described by Jentzsch et al. [14] for serum samples. Serum TBARS were expressed as nM malondialdehyde (MDA)/mL. The determination of the nitrite/nitrate (NO_x_) levels was carried out using the method of reducing nitrate to nitrite with the Griess reagent; the measurement of the reaction was carried out at 550 nm using sodium nitrate as standard. Levels of carbonyl protein in plasma were determined in supernatants, as described by Reznick and Packer [15]. Results were expressed as nmol of carbonyl formed/mg of protein. Levels of ROS in plasma were determined by the 2′,7′-dichlorofluorescein diacetate (DCF-DA) oxidation method as described by LeBel et al. [16] using excitation and emission of the wavelengths of 485 and 538 nm, respectively. Results were expressed as U DCF/mL.

Glutathione S-transferase (GST) activity in serum was measured based on the method described by Habig et al. [17]. Enzymatic activity was expressed as U GST/mg of protein. Glutathione peroxidase (GPx) activity in serum was measured according to the methodology described by Paglia and Valentine [18]. Enzymatic activity was expressed as U GPx/mg of protein. Catalase activity (CAT) was measured in red blood cell hemolysate using the method of Beers and Sizer [19], with the decomposition of H_2_O_2_ directly followed by the decrease in absorbance at 240 nm and the difference in absorbance per min/mg of Hb was taken as a measure of CAT activity. The evaluation of superoxide dismutase (SOD) activity was carried out in blood according to the method described by Misra and Fridovich [20]; this method is based on the inhibition of epinephrine autoxidation at pH 10.2 by SOD.

### 2.10. Statistical Analysis

All data were analyzed using the SAS MIXED procedure (SAS Inst. Inc., Cary, NC, USA; version 9.4), with the Satterthwaite approximation to determine the denominator degrees of freedom for the fixed effects test. All variables (productive, biochemistry, blood count, protein count, and oxidants) were analyzed as repeated measures and tested for fixed effects of treatment, day, and treatment × day. All results obtained on d1 for each variable were also included as covariants; however, the command for covariates was removed from the model when *p* > 0.05. Averages were determined using PDIFF, and all results were reported as LSMEANS followed by the standard error. Significant differences were defined when *p* ≤ 0.05 and trends when *p* > 0.05 and >0.10.

## 3. Results

Infected animals showed an increase in milk production after treatment (*p* < 0.01), as shown in Table 1; however, dry matter consumption was not affected, with no differences being observed between positive and negative animals on the day of treatment (day 1) with the same occurring ten days after treatment.

When we analyzed the effect of *T. vivax* on the white cells, we observed leukopenia, lymphopenia, and a reduction in the number of granulocytes when studying the interaction of days versus treatment (Table 2). No effects were observed on hematological parameters when studying the interaction between day 1 versus day 10 post-treatment. No difference was observed concerning monocytes in the interactions of days versus treatment and between days of the experiment. Another effect observed in the day versus treatment interaction, demonstrated in Table 2, was the reduction in the erythrocyte count, confirming an anemic condition in the infected animals (*p* < 0.01), which was accompanied by a reduction in hemoglobin (mg/dL), and a reduction in hematocrit (%), and fall in the platelets count (*p* < 0.01).

The infection also affected biochemical parameters, with an effect of day versus treatment on C-reactive protein, creatine kinase, and cholinesterase (Table 3), major in infected cows. For total protein, globulins, GGT, and AST, an interaction was demonstrated between treatment versus day, with this effect (high in seropositive cows) being maintained in the analysis between day 1 and day 10 post-treatment (Table 3). Serum calcium levels were lower in seropositive cows on day 1; however, ten days after treatment, there was no longer any difference between groups. There was no effect on glucose, cholesterol, fructosamine, creatine, albumin, urea, and magnesium levels.

There was an effect on serum proteins (Table 4), with an increase in the levels of IgA, heavy chain Ig, serum amyloid, ceruloplasmin, ferritin, and haptoglobin, and a reduction in transferrin levels in seropositive cows was observed. Day effect was observed for haptoglobin levels in infected cows, with a difference between days 1 and 10.

Results of markers of oxidative stress were demonstrated in Table 5. Cows diagnosed with trypanosomosis presented high levels of reactive oxygen species, lipid peroxidation, protein oxidation, nitrite/nitrate, superoxide dismutase, and glutathione peroxidase activity. Catalase and GST showed lower activity in seropositive cows’ blood than the control on the day of diagnosis, which was no longer observed ten days after treatment. Effect of day and treatment x day interaction (day 10) was observed for total antioxidants in serum, with FRAP levels being higher in seropositive cows.

## 4. Discussion

The present work demonstrated the effect of *T. vivax* infection in Jersey cows and the responses on productive, hematologic, proteinogram, and oxidative stress. A reduction in the production of infected animals was observed but without changes in feed intake (Table 1), which demonstrates the redirection of nutrients to support the immune response, like the activation response of the immune system by the infusion of lipopolysaccharides [21]. Activating the immune response in trypanosomosis is essential in controlling the disease and developing pathological changes. In African trypanosomosis the beginning of the infection, the activation of macrophages (predominantly M1), and the production of inflammatory cytokines is essential for controlling parasitemia, however, the persistence of the M1 type and the activation of the mononuclear phagocyte system is associated with anemia resulting from increased erythrophagocytosis [22,23]. The present work demonstrates the reduction in the erythrocyte count below 5 × 10^6^ µL, characterizing an anemic condition (Table 2) [24] However, the role of erythrophagocytosis and sialidases in the development of anemia was not tested in this work.

When we analyze the hematological response to the infection, the reduction in the count of lymphocytes and granulocytes is notable, but without a statistical difference between the count of monocytes (precursor of macrophages after tissue migration), as shown in Table 2. Studies show that the production of cytokines is essential and modulated by the type of cellular response represented at the beginning of the infection by the increase in inflammatory cytokines and transition to anti-inflammatory cytokines with IL-10, for example [25]. Another observed effect of these cytokines (IL-10) is the increase in ferritin and the reduction in the number of platelets (thrombocytopenia) [22]. The data presented here demonstrated the reduction in platelet count and the increase in ferritin levels, together with the increase in ceruloplasmin and the reduction in transferrin. An increase in these proteins is a component of the acute phase response, accompanied by an increase in proteins such as haptoglobin, SAA, and C-reactive protein, a consequence of the increase in their hepatic synthesis stimulated by pro-inflammatory cytokines [26]. The vascular effect of the infection must also be considered in explaining the reduction in platelets, with vascular damage and coagulopathies observed in *T. vivax* infection in vessels of the brain, heart, lung, liver, spleen, pancreas, kidneys, and adipose tissue in experiments with mice [6,7].

Sampaio et al. [26] demonstrated an increase in the levels of immunoglobulin and acute phase proteins, which corroborates the data from the present experiment where we observed high levels of total proteins, globulins, IgA, and heavy chain Ig, a result of the activation of lymphocytes B and development of the humoral response to *T. vivax* infection [25]. As observed in this study, the infection of *T. vivax* leads to hypocalcemia, possibly due to increased vascular permeability and calcium sequestration into the interstitial fluid. Another possible route is stimulating the Ca-sensing receptor in the parathyroid by inflammatory cytokines; however, this pathway is not fully elucidated in cattle [27] and the mechanism of calcium depletion is not the focus of this study.

Gamma-glutamyl transferase and AST levels indicate hepatobiliary injury, liver injury, muscle injury, or hemolysis, respectively, with creatine kinase (CK) being a reliable marker for muscle injury [28]. The present work demonstrated that *T. vivax* infection leads to increased GGT, AST, and CK levels, being suggestive of liver lesions and destruction of red blood cells that contribute to the observed anemia and muscle damage. Studies with *T. vivax* showed vascular lesions in cardiac muscle [7], corroborating an increase in CK levels but without changes in creatinine levels; however, skeletal muscle injuries must be considered in explaining the increase in creatine kinase.

Cholinesterase, a marker of low-grade inflammation and oxidative stress can indicate cellular injury [29]. The present work demonstrated an increase in cholinesterase levels (Table 3), indicating inflammation, whose presence is corroborated by the strong response of acute phase proteins. FRAP levels, an indicator of the oxidation-reduction capacity of plasma iron and a marker of “antioxidant capacity”, were low and associated with high levels of TBARS, carbonyl, and serum ROS, demonstrated in this experiment that indicates lipid peroxidation, protein oxidation, and increased in circulating reactive oxygen species, showing oxidative stress and cellular damage [30].

In natural infection by *T. evansi* in camels, an increase in CAT and SOD was observed [31]; however, in cattle, infection by *T. vivax* reduces serum CAT levels and did not change SOD levels. Elevated levels of nitric oxide were observed, and its increase has positive effects on the control of parasitemia in *T. evansi* infections; however, it is related to the chronic phase of the infection with a positive relationship with anemia and actively acting on oxidative stress [23]. An increase in GPx, responsible for the reduction of hydrogen peroxide, and the reduction of GST, which catalyzes the conjugation of electrophilic substances to glutathione, was also observed, demonstrating an increase in reactive oxygen species [32] and a specific oxidative response pattern for *T. vivax* infection in Jersey cows.

## 5. Conclusions

Natural infection by *T. vivax* in Jersey cows interferes with milk production, which is accompanied by leukopenia and anemia, a reduction in lymphocytes and granulocytes, and a reduction in platelet counts without altering the monocyte count. An acute response was observed, marked for effect on serum proteins, the effect of the inflammatory response, and iron metabolism resulting from *T. vivax* infection, with an increase in enzymes related to liver and muscle damage. It has been shown that trypanosome infection triggers the production of ROS and NO_x_, with increased lipid peroxidation and protein oxidation, together with a peculiar pattern of response of antioxidant enzymes and redox metabolism. Treatment with isometamidium chloride combined with liver protector and vitamin B12 was effective in treating cows against *T. vivax*.

## Figures and Tables

**Table 1:** Milk production and consumption of cows are positive and negative for infection of *Trypanosoma vivax*, as well as the effects of treatment of isometamidium on day and interaction of treatment and days on milk production and feed intake.

Variable	Positive (n = 14)	Negative (n = 6)	SEM	*p*—Treat ^#^	*p*—Treat × Day *
Milk production, kg				0.91	0.17
d1 (diagnostic)	17.1	17.4	0.52		
d5	17.5	17.3	0.51		
d10	18.8	17.4	0.51		
Mean 1 to 10	17.9	17.4	0.50		
Increase production (1 to 10), kg	1.72 ^a^	0.0 ^b^	0.05	0.01	-
Feed intake, kg of DM				0.89	0.62
d1	10.1	10.5	0.03		
d5	10.4	10.5	0.03		
d10	10.5	10.5	0.03		
Total intake	100.2	105.6	0.14		

^#^ Shows treatment effect, where different letters on the same line show significant differences (*p* ≤ 0.05) and trends (*p* ˃ 0.05 and ≤0.1) between groups; * Shows treatment × day interaction, where different lowercase letters (^a,b^) on the same line show significant differences (*p* ≤ 0.05) and trends (*p* ˃ 0.05 and ≤0.1) between groups.

**Table 2:** Effects of *T. vivax* infection of Blood count, White blood cell count, platelets, and interaction on day and treatment and effects on day 1 versus day 10 post-treatment.

Variable	Positive	Negative	SEM	*p*—Treat × Day *	*p*—Day ^+^
Leukocytes (×10^3^ cel/µL)				0.01	0.72
d1	4.25 ^b^	7.82 ^a^	0.28		
d10	4.68 ^b^	7.74 ^a^	0.23		
Lymphocytes (×10^3^ cel/µL)				0.01	0.81
d1	1.58 ^b^	3.98 ^a^	0.41		
d10	2.04 ^b^	4.06 ^a^	0.36		
Monocytes (×10^3^ cel/µL)				0.62	0.78
d1	0.68	0.89	0.24		
d10	0.54	0.94	0.29		
Granulocytes (×10^3^ cel/µL)				0.05	0.69
d1	1.99 ^b^	2.95 ^a^	0.22		
d10	2.10	2.74	0.26		
Erythrocytes (×10^6^ cel/µL)				0.01	0.22
d1	4.62 ^b^	6.25 ^a^	0.06		
d10	4.97 ^b^	6.12 ^a^	0.05		
Hemoglobin (mg/dL)				0.01	0.36
d1	8.96 ^b^	12.0 ^a^	0.15		
d10	9.21 ^b^	12.4 ^a^	0.13		
Hematocrit (%)				0.01	0.31
d1	22.8 ^b^	29.7 ^a^	0.64		
d10	24.2 ^b^	30.8 ^a^	0.52		
Platelets (×10^3^ µL)				0.01	0.71
d1	255 ^b^	375 ^a^	9.74		
d10	246 ^b^	351 ^a^	8.58		

* Shows treatment × day interaction, where different lowercase letters (^a,b^) on the same line show significant differences (*p* ≤ 0.05) and trends (*p* ˃ 0.05 and ≤0.1) between groups; ^+^ Shows day effect.

**Table 3:** Clinical biochemistry parameters for infected (positive) and negative cows. Interactions of day versus treatment and day 1 versus day 10 post-treatment.

Variables	Positive	Negative	SEM	*p*—Treat × Day *	*p*—Day ^+^
Glucose (mg/dL)				0.92	0.86
d1	79.5	82.3	2.65		
d10	73.9	75.6	2.67		
CK (U/L)				0.01	0.11
d1	551 ^a^	288 ^b^	18.2		
d10	647 ^a^	269 ^b^	17.5		
Cholesterol (mg/dL)				0.54	0.95
d1	151	163	3.95		
d10	153	160	3.90		
Fructosamine (mg/dL)				0.75	0.92
d1	2.39	2.30	0.25		
d10	2.34	2.31	0.19		
Creatinine (mg/dL)				0.87	0.97
d1	0.58	0.53	0.06		
d10	0.55	0.51	0.07		
Total protein (g/dL)				0.01	0.05
d1	9.05 ^aA^	7.10 ^b^	0.32		
d10	8.25 ^B^	7.33	0.28		
Albumin (g/dL)				0.68	0.98
d1	3.10	3.05	0.12		
d10	3.04	2.94	0.11		
Urea (mg/dL)				0.92	0.96
d1	33.8	31.3	1.74		
d10	34.6	33.0	1.72		
Globulin (g/dL)				0.01	0.05
d1	5.94 ^aA^	4.04 ^b^	0.26		
d10	5.20 ^B^	4.38	0.26		
GGT (U/L)				0.01	0.02
d1	49.2 ^aA^	24.8 ^b^	3.05		
d10	32.4 ^B^	25.0	3.12		
AST (U/L)				0.01	0.01
d1	134 ^aA^	96.9 ^b^	4.16		
d10	96.0 ^B^	83.6	4.19		
Cholinesterase				0.01	0.28
d1	120 ^a^	92.7 ^b^	3.54		
d10	134 ^a^	84.7 ^b^	3.28		
Calcium				0.02	0.02
d1	8.03 ^bA^	9.53 ^a^	0.25		
d10	8.75 ^B^	9.68	0.25		
Magnesium				0.11	0.96
d1	2.35	2.76	0.09		
d10	2.32	2.33	0.10		
C-reactive protein				0.05	0.64
d1	0.29 ^a^	0.26 ^b^	0.01		
d10	0.28	0.28	0.01		

* Shows treatment × day interaction, where different lowercase letters (^a,b^) on the same line show significant differences (*p* ≤ 0.05) and trends (*p* ˃ 0.05 and ≤0.1) between groups; ^+^ Shows day effect, where different capital letters (^A,B^) on the same column show significant differences (*p* ≤ 0.05) between days 1 to 10; Abbreviations: CK = creatine kinase; GGT = Gamma glutamyl transferase; AST = aspartate aminotransferase.

**Table 4:** Proteinogram of cows infected and not infected by *T. vivax*. Interactions between day and treatment and day 1 versus day 10 post-treatment.

Variables	Positive	Negative	SEM	*p*—Treat × Day *	*p*—Day ^+^
IgA (g/dL)				0.01	0.35
d1	1.32 ^a^	0.64 ^b^	0.08		
d10	1.38 ^a^	0.71 ^b^	0.08		
Heavy chain Ig (g/dL)				0.01	0.68
d1	1.91 ^a^	1.03 ^b^	0.09		
d10	1.86 ^a^	0.97 ^b^	0.08		
Soro amiloride				0.04	0.97
d1	0.35 ^a^	0.28 ^b^	0.02		
d10	0.34	0.29	0.02		
Ceruloplasmin (g/dL)				0.01	0.95
d1	0.91 ^a^	0.56 ^b^	0.04		
d10	0.93 ^a^	0.58 ^b^	0.05		
Haptoglobin (g/dL)				0.01	0.01
d1	0.45 ^aA^	0.21 ^b^	0.02		
d10	0.32 ^B^	0.23	0.03		
Ferritin (g/dL)				0.01	0.80
d1	0.32 ^a^	0.18 ^b^	0.02		
d10	0.35 ^a^	0.19 ^b^	0.02		
Transferrin (mg/dL)				0.01	0.29
d1	0.21 ^b^	0.45 ^a^	0.04		
d10	0.25 ^b^	0.44 ^a^	0.05		

* Shows treatment × day interaction, where different lowercase letters (^a,b^) on the same line show significant differences (*p* ≤ 0.05) and trends (*p* ˃ 0.05 and ≤0.1) between groups; ^+^ Shows day effect, where different capital letters (^A,B^) on the same column show significant differences (*p* ≤ 0.05) between days 1 to 10.

**Table 5:** Oxidative status of cows positive and negative for infection of *T. vivax*. Interaction of treatment versus day and day 1 versus day 10 post-treatment.

Variables	Positive	Negative	SEM	*p*—Treat × Day *	*p*—Day ^+^
TBARS in serum (nmol/mL)				0.01	0.05
d1	25.9 ^aA^	14.6 ^b^	4.12		
d10	18.7 ^B^	14.1	3.13		
ROS in serum				0.01	0.01
d1	111 ^aA^	62.7 ^b^	18.2		
d10	76.1 ^B^	58.4	9.75		
Carbonyl in serum				0.01	0.88
d1	6.85 ^a^	2.74 ^b^	1.69		
d10	6.59 ^a^	3.08 ^b^	1.45		
NO_x_ in serum				0.01	0.92
d1	86.2	55.1	7.52		
d10	81.3	58.3	7.39		
GST in serum				0.01	0.01
d1	232 ^aB^	414 ^a^	19.8		
d15	430 ^A^	417	15.5		
GPx in serum				0.01	0.82
d1	21.8 ^a^	12.7 ^b^	2.85		
d10	25.2 ^a^	14.3 ^b^	2.74		
SOD in blood				0.76	0.62
d1	5.98 ^a^	3.05 ^b^	0.54		
d10	6.24 ^a^	3.16 ^b^	0.55		
CAT in blood				0.01	0.01
d1	2.69 ^bB^	8.47 ^a^	0.68		
d10	6.87 ^A^	7.96	0.71		
FRAP in serum				0.01	0.01
d1	62.6 ^B^	68.4	5.96		
d10	95.6 ^aA^	70.3 ^b^	4.99		

* Shows treatment × day interaction, where different lowercase letters (^a,b^) on the same line show significant differences (*p* ≤ 0.05) and trends (*p* ˃ 0.05 and ≤0.1) between groups; ^+^ Shows day effect, where different capital letters (^A,B^) on the same column show significant differences (*p* ≤ 0.05) between days 1 to 10; Abbreviations: TBARS = Thiobarbituric acid reactive substances; ROS = reactive oxygen species; NO_x_ = Nitrite/nitrate; GST = Glutathione S-transferases; GPx = Glutathione peroxidase; SOD = Superoxide dismutase; CAT = catalase; FRAP = ferric reducing antioxidant ability.

## Data Availability

The data are with the authors and may be made available upon request.
